# Robustness Evaluation of a Deep Learning Model on Sagittal and Axial Breast DCE-MRIs to Predict Pathological Complete Response to Neoadjuvant Chemotherapy

**DOI:** 10.3390/jpm12060953

**Published:** 2022-06-10

**Authors:** Raffaella Massafra, Maria Colomba Comes, Samantha Bove, Vittorio Didonna, Gianluca Gatta, Francesco Giotta, Annarita Fanizzi, Daniele La Forgia, Agnese Latorre, Maria Irene Pastena, Domenico Pomarico, Lucia Rinaldi, Pasquale Tamborra, Alfredo Zito, Vito Lorusso, Angelo Virgilio Paradiso

**Affiliations:** 1 Struttura Semplice Dipartimentale di Fisica Sanitaria, I.R.C.C.S. Istituto Tumori “Giovanni Paolo II”, Viale Orazio Flacco 65, 70124 Bari, Italy; r.massafra@oncologico.bari.it (R.M.); m.c.comes@oncologico.bari.it (M.C.C.); s.bove@oncologico.bari.it (S.B.); v.didonna@oncologico.bari.it (V.D.); d.pomarico@oncologico.bari.it (D.P.); p.tamborra@oncologico.bari.it (P.T.); 2 Dipartimento di Medicina di Precisione Università della Campania “Luigi Vanvitelli”, 80131 Naples, Italy; gianluca.gatta@unicampania.it (G.G.); a.latorre@oncologico.bari.it (A.L.); 3 Unità Operativa Complessa di Oncologia Medica, I.R.C.C.S. Istituto Tumori “Giovanni Paolo II”, Viale Orazio Flacco 65, 70124 Bari, Italy; f.giotta@oncologico.bari.it (F.G.); vitolorusso@me.com (V.L.); 4 Struttura Semplice Dipartimentale di Radiologia Senologica, I.R.C.C.S. Istituto Tumori “Giovanni Paolo II”, Viale Orazio Flacco 65, 70124 Bari, Italy; 5 Unità Operativa Complessa di Anatomia Patologica, I.R.C.C.S. Istituto Tumori “Giovanni Paolo II”, Viale Orazio Flacco 65, 70124 Bari, Italy; m.pastena@oncologico.bari.it (M.I.P.); a.zito@oncologico.bari.it (A.Z.); 6 Struttura Semplice Dipartimentale di Oncologia Per la Presa in Carico Globale del Paziente, I.R.C.C.S. Istituto Tumori “Giovanni Paolo II”, Viale Orazio Flacco 65, 70124 Bari, Italy; l.rinaldi@oncologico.bari.it; 7 Oncologia Sperimentale e Biobanca, I.R.C.C.S. Istituto Tumori “Giovanni Paolo II”, Viale Orazio Flacco 65, 70124 Bari, Italy; a.paradiso@oncologico.bari.it

**Keywords:** pathological complete response, early prediction, magnetic resonance imaging, deep learning

## Abstract

To date, some artificial intelligence (AI) methods have exploited Dynamic Contrast-Enhanced Magnetic Resonance Imaging (DCE-MRI) to identify finer tumor properties as potential earlier indicators of pathological Complete Response (pCR) in breast cancer patients undergoing neoadjuvant chemotherapy (NAC). However, they work either for sagittal or axial MRI protocols. More flexible AI tools, to be used easily in clinical practice across various institutions in accordance with its own imaging acquisition protocol, are required. Here, we addressed this topic by developing an AI method based on deep learning in giving an early prediction of pCR at various DCE-MRI protocols (axial and sagittal). Sagittal DCE-MRIs refer to 151 patients (42 pCR; 109 non-pCR) from the public I-SPY1 TRIAL database (DB); axial DCE-MRIs are related to 74 patients (22 pCR; 52 non-pCR) from a private DB provided by Istituto Tumori “Giovanni Paolo II” in Bari (Italy). By merging the features extracted from baseline MRIs with some pre-treatment clinical variables, accuracies of 84.4% and 77.3% and AUC values of 80.3% and 78.0% were achieved on the independent tests related to the public DB and the private DB, respectively. Overall, the presented method has shown to be robust regardless of the specific MRI protocol.

## 1. Introduction 

Neoadjuvant chemotherapy (NAC) is a form of oncological therapy that is used to reduce the size of the tumor and the infiltration of extra-glandular structures, consequently enabling a more conservative surgery [1,2]. Over the last few years, the adoption of NAC is growing, especially against some molecular subtypes, such as triple-negative tumors and HER2+ tumors of any size, as there is concrete evidence of greater clinical efficacy [3,4,5]. The achievement of the pathological Complete Response (pCR) at the end of NAC, namely, the absence of residual invasive disease or metastatic lymph nodes at the end of the entire course of the therapy, which is assessed by pathologists on the excised tissue after surgery, leads to a more favorable prognosis than traditional treatments, with an increase in disease-free survival and overall survival [3,6,7,8]. The prediction of NAC outcome before the beginning of the therapy (early prediction) in terms of pCR is a hot topic in the current clinical research since an earlier identification of patients who will effectively respond to NAC is crucial to improve and change treatment planning during the course of treatment, optimize costs and spare such patients from potentially ineffective or toxic chemotherapy [9,10]. Not less relevant, patients who are identified as responders to NAC since the beginning of the therapy, are more likely to take advantage from breast conserving surgery, avoiding a full mastectomy [1]. Dynamic Contrast-Enhanced Magnetic Resonance Imaging (DCE-MRI) is one of the medical imaging techniques that have come to play a prominent role in the radiomic framework for breast cancer. The possibility of evaluating the same findings with several sequences and in several planes (axial, sagittal, and coronal) allows us to better identify the characteristics of the lesion and the relationships with the contiguous structures [11,12]. Breast MRIs are mostly performed and interpreted in axial and sagittal planes, since coronal projections require more slices and are affected by a greater number of motion artifacts due to breathing [13]. Axial acquisition (a horizontal plane with respect to the human body, dividing it into upper and lower parts) is usually faster than sagittal acquisition (a longitudinal plane with respect to the human body, dividing it into left and right parts). Axial acquisition also provides a better overview of both breasts simultaneously [14]. However, there is no universal guideline for slice direction selection in breast MRI scanning: MRI acquisition protocols can vary across cancer institutions worldwide, with some institutions not conducting the acquisition of both axial and sagittal projections, but instead only one of the two. To date, the evaluation of the NAC response has been successfully investigated through the analysis of DCE-MR images [9,10] since the application of computerized algorithms on MRIs has proven to be crucial in highlighting morphological characteristics of the lesion, tumor size, and even residual tumor [15]. Thus, a higher accuracy in evaluating NAC response has emerged with respect to other imaging techniques [16,17]. In the state-of-the-art, some studies focused on MRI-based radiomics have exploited tissue, peritumoral, or intratumoral features in combination with histopathological information to give an early prediction of pCR, i.e., prior to therapy or during the earliest stages of the therapy [18,19,20,21,22], with the final goal of optimizing treatment planning for each individual patient. However, these kinds of features, which are extracted from raw images, are handcrafted by experts in the field and could be influenced by human bias. More recently, methods based on deep learning, a branch of artificial intelligence (AI), have been designed to automatically extract relevant features from images, including MRIs, without human intervention. The task of “early” prediction of pCR to NAC has been achieved through such deep learning techniques exploiting algorithms, known as Convolutional Neural Networks (CNNs), which resemble the neuron functions of the human brain. Compared to handcrafted features, the exploitation of CNNs to extract features from images has proven to be more promising [23]. MRI examinations acquired prior to treatment (baseline MRI) or at initial stages of NAC, e.g., after the initial cycles, have been used as input data for CNNs [24,25,26,27,28]. As far as we know, the methods that have already proposed to predict pCR to NAC in breast cancer patients have been developed and tailored either for sagittal or axial MRI acquisition views. Hence, more generalizable methods, rather than protocol-specific approaches, need to be developed with the aim of being easily utilized in large multi-institutional studies where the acquisition protocols may differ across diverse institutions. In this work, an AI approach based on deep learning and specifically on a pre-trained CNN has been developed and separately applied on both baseline axial and sagittal pre-treatment MRI examinations to give an early prediction of pCR in breast cancer patients undergoing NAC. More in detail, the developed model wants to give a prediction of the outcome of NAC in terms of pCR before the beginning of the therapy. The robustness of the approach at various MRI acquisition views, i.e., sagittal and axial views, in reference to a public DB and a private DB has been evaluated. Finally, this study is a first effort to pave the way to the design of an effective approach that could, on the one hand, be able to predict pCR to NAC “early on” in breast cancer patients and, on the other hand, represent a flexible predictive tool to be easily utilized in clinical practice across diverse institutions in accordance with their own imaging acquisition protocols.

## 2. Materials and Methods

### 2.1. Data Collection

A binary classification task was developed for an early prediction of breast cancer patients who have, or have not, achieved pCR to NAC and whose classifications were indicated as pCR and non-pCR, respectively. The term pathological complete response indicates the absence of residual invasive disease or metastatic lymph nodes at the completion of the entire course of treatment. It is evaluated at the end of chemotherapy and after surgery. For the intended purpose, pre-treatment DCE-MRI examinations were analyzed. Specifically, we defined an AI framework and evaluated its performance to give an early prediction of pCR on two different case studies that dealt with DCE-MRI examinations of breast cancer patients undergoing NAC: a public DB containing sagittal DCE-MRIs and a private DB consisting of axial DCE-MRIs. The public DB, entitled Investigation of Serial Studies to Predict Your Therapeutic Response with Imaging and Molecular Analysis (I-SPY1 TRIAL) [6,29,30], is available online on The Cancer Imaging Archive (https://wiki.cancerimagingarchive.net; accessed on 25 November 2021) [31] and includes cases of 230 women with breast tumors of at least 3 cm in size. They were recruited between 2002 and 2006 and underwent NAC according to an anthracycline–cyclophosphamide (AC) regimen either on its own or followed by taxane. Sagittal MRI examinations at different timepoints were obtained. At each timepoint, three images were acquired using 1.5 T field-strength MR imaging systems: a single pre-contrast image and two images taken approximately 2 min and 7 min post contrast injection. Among all the subjects, a set of 151 patients (42 pCR; 109 non-pCR) was then considered, as they had undergone an MRI scan prior to treatment. The private DB consists of a set of 74 patients who were registered as having a first breast tumor diagnosis between 2018 and 2021 at Istituto Tumori “Giovanni Paolo II” in Bari (Italy) and received NAC. Among them, 36 patients were treated with AC followed by taxane; 7 patients underwent AC followed by taxane and trastuzumab; 2 patients received taxane and trastuzumab; 4 patients followed a regimen consisting of AC, pertuzumab, trastuzumab and taxane; 15 patients were treated with pertuzumab, trastuzumab and taxane; and 3 patients followed other treatment regimens. Axial MRI examinations at different timepoints were performed. At each timepoint, six images were acquired in the prone position with a dedicated seven-channel breast coil on a 1.5 Tesla PHILIPS scanner (Achieva, Philips Medical Systems, Amsterdam, The Netherlands): a single pre-contrast image and five images corresponding to approximately each minute post contrast injection, respectively. An MRI examination prior to treatment was acquired for all 74 patients (22 pCR; 52 non-pCR).

### 2.2. Data Pre-Processing

**Figure 1:**
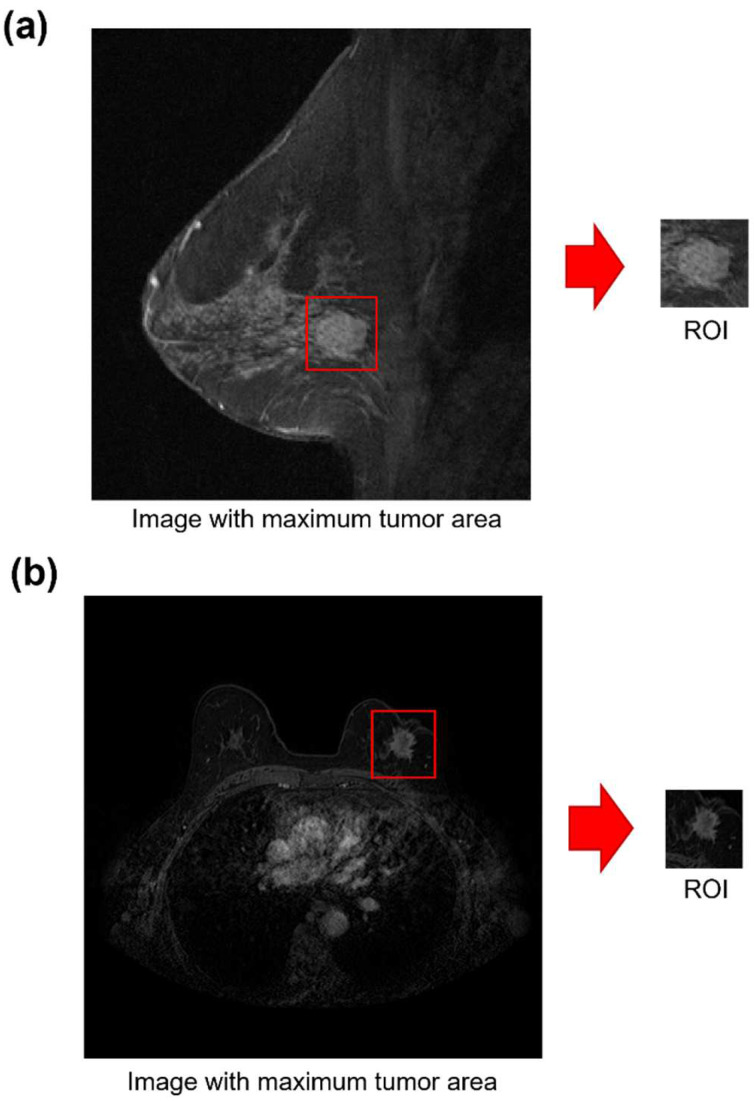
Examples of baseline MR image acquired according to (**a**) a sagittal view of a public DB patient and (**b**) an axial view of a private DB patient.

The baseline MRI examination at around the second minute post contrast injection was processed for both datasets. Since previous studies have underlined that the peritumoral region may benefit from a more accurate prediction of pCR in breast cancer patients undergoing NAC [20,27], a semi-automatic algorithm to define a Region Of Interest (ROI) also including the peritumoral zone was developed for all the images belonging to the two case studies: for each patient, an ROI around the center of the tumor mass and containing both intratumoral and peritumoral regions from the MRI scan with the largest tumor area was identified. All the ROIs were reviewed by our expert breast imaging radiologist with over 20 years of experience. Examples of sagittal and axial acquisition from the public DB and private DB with their corresponding ROIs are shown in Figure 1a,b, respectively.

### 2.3. Statistical Analysis

Some clinical variables were included in the information available online on the public DB: age, ER, PgR, HER2 and tumor size (T), which is the largest diameter of the lesion evaluated from the baseline MRI examination. We were provided with two other variables by the authors of the public DB: Ki67 and grading. The same variables were considered for the private DB. The relationship between each clinical feature and the pCR value (0 for the patients belonging to the non-pCR classification and 1 for the patients belonging to the pCR classification) was evaluated by means of an overall statistical test on the datasets independently: the Wilcoxon–Mann–Whitney test [32] was used for continuous features, and the Spearman rank test [33] was used for features measured on an ordinal scale. A result was considered statistically significant when the *p*-value was less than 0.10.

### 2.4. An AI Framework to Predict pCR “Early On” from Baseline MRI Examinations

**Figure 2:**
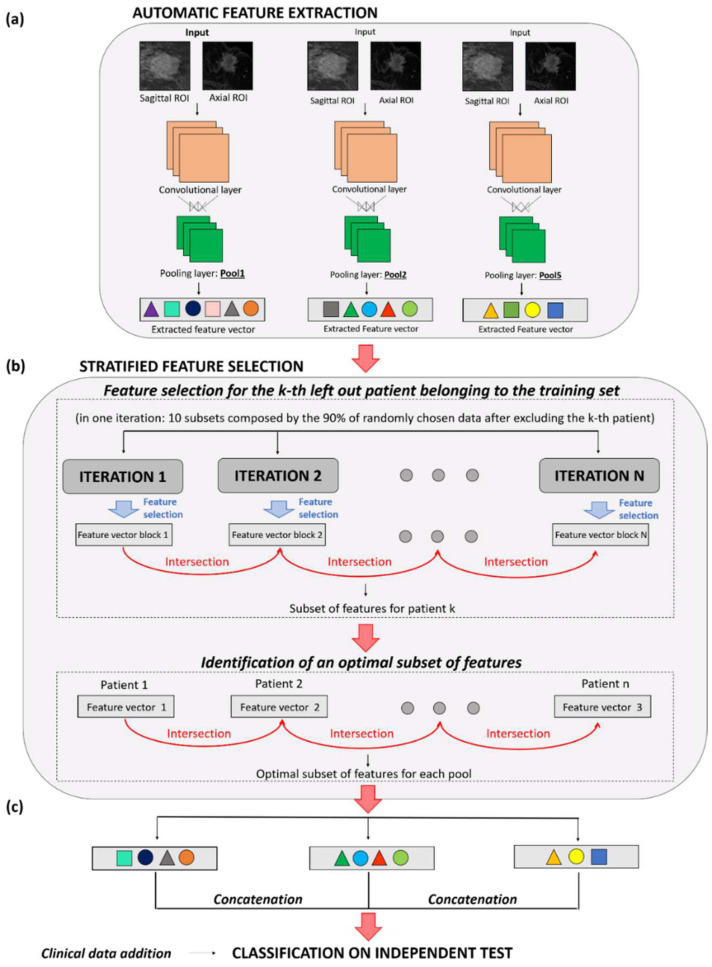
Workflow of the proposed AI framework for early pCR prediction. The approach consists of three main steps: (**a**) Automatic feature extraction through a pre-trained CNN; (**b**) Stratified feature selection; (**c**) Classification on the independent test. The method has been applied on sagittal and axial baseline MRIs separately.

The main steps of the proposed method are briefly reported in the following and represented in Figure 2. The method was trialed on a set of patients (training set) and then validated on a set of patients (independent test set) identified on both the two databases separately. Specifically, the two datasets were divided into training and test sets (70% and 30% of patients, respectively) according to random stratified sampling. In this section, the main details of the method have been reported. Please refer to Appendix A for a more detailed description of the methods used. Features were automatically extracted from three different layers (called *pool1*, *pool2* and *pool5*) of a pre-trained Convolutional Neural Network (CNN), named AlexNET and firstly introduced by A. Krizhevsky and his collegues [34] (Figure 2a). A stratified feature selection process was then developed to identify the most stable CNN features.

Such features were identified in 70% of the overall data for the two databases (training sets), i.e., 106 patients (29 pCR; 77 non-pCR) from the public DB and 52 patients (15 pCR; 37 non-pCR) from the private DB. First, a set of optimal CNN-extracted features was obtained in correspondence with each of the three layers (Figure 2b). Next, a feature concatenation was conducted to merge the optimal features related to the three different layers: an optimal set of features, named F-merged, was constructed. With the aim of validating the proposed approach, the remaining 30% of patients on the two databases was used to define two independent tests, one for each of the case studies (Figure 2c). A total of 45 patients (13 pCR; 32 non-pCR) formed the independent test for the public DB, whereas the test for the private DB was composed of 22 patients (7 pCR; 15 non-pCR). An SVM classifier was built by exploiting the F-merged feature set alone or in combination with some clinical variables. The performances of these classifiers were compared with an SVM classifier using the clinical variables alone. The performance achieved by the designed classifiers was measured in terms of the Area Under the Curve (AUC), the Receiver Operating Characteristic (ROC) curve and other standard metrics, such as accuracy, sensitivity and specificity. ‘Accuracy’ evaluates the rate of correct classification between the groups of patients who have achieved pCR or not. ‘Sensitivity’ and ‘specificity’ measure the proportion of pCR and non-pCR subjects who were correctly identified. AUC, instead, indicates the ability of the classifier to correctly assign patients to the two classes (pCR and non-pCR) by assuming values ranging from 50% (meaning random guessing) to 100% (meaning perfect separability). Each patient with a classification score exceeding a threshold determined by the ratio of the number of patients belonging to the pCR-class over the overall number of patients comprising the dataset [35], i.e., 0.28 for the public DB and 0.30 for the private DB, was assigned to the pCR-class. All the steps of our analysis were performed by using the MATLAB R2019a (MathWorks, Inc., Natick, MA, USA) software.

## 3. Results

### 3.1. Statistical Analysis Results

In Table 1, the clinical characteristics of patients belonging to the public DB and private DB are split into patients who have achieved pCR (pCR, 42 and 22 for the public DB and private DB, respectively) and patients who have not achieved pCR (non-pCR, 109 and 52 for the public DB and private DB, respectively). The rate of patients who achieved pCR (pCR) was very similar between the two case studies, i.e., they corresponded to 28% and 30% of the public DB and the private DB, respectively. The public DB contains the ER and PgR variables as categorical features. Specifically, they were binary features assuming a negative value (0) or a positive value (1). They were determined by immunohistochemistry (IHC) and were considered positive if the Allred score was ≥3 [36,37]. The grading variable was evaluated according to the SBR/Elston Classification and the Ki67 IHC staining was performed at the University of North Carolina by using the standard avidin–biotin complex technique. Four classes representing four different levels of Ki67 were recognized [36]: negative if Ki67 was equal to 0; low if Ki67 was less than 10%; intermediate if the variable was between 10% and 25%; and high if Ki67 was greater than 25%. The HER2 variable was evaluated according to IHC and/or fluorescent in situ hybridization assays. The T variable expressing the largest diameter of the lesion within the sagittal MRI examination was part of the clinical information available online [31].

In our DB, the variables ER (Clone EP1 DAKO), PgR (Clone PgR636) and Ki67 (Clone MIB1 DAKO) were evaluated in percentage values; the grading values were assessed according to Elston Classification, whereas the HER2 (polyclonal Rabbit Anti-Human c-erb 2 Oncoprotein) variable was measured according to the ASCO-CAP guidelines. For the sake of a fair comparison between the two case studies, the percentage values of ER and PgR being compared in our DB were converted into categorical binary variables (negative if ER and PgR were equal to 0; positive if ER and PgR assumed values greater than or equal to 1%). Similarly, the Ki67 value was converted in the same categories as the public DB. In this case, the T variable represented the maximum diameter of the lesion evaluated and reported by our radiologist during the axial MRI scan acquisition.

Table 2 summarizes the *p*-values obtained by performing an association test between pCR and therapy and each clinical factor. No significant association was observed between the couples age–pCR and T–pCR for either of the two case studies (*p*-value > 0.10). Significant associations (*p* < 0.05) emerged between pCR and the histological variables ER, PgR and grading for both the case studies. Moreover, pCR and HER2 showed a significant association, although less significance was found in the private DB (*p*-value < 0.10). However, this was acceptable given the reduced sample size. It is worth noting that the Ki67 was closely associated with pCR only for the public DB (*p*-value < 0.05).

By considering all the patients from the private DB, who underwent different types of therapy pathways, no significant association between Ki67 and pCR emerged (*p*-value > 0.10). However, when the statistical test was performed only on patients of the private DB who underwent the AC + taxane scheme, a statistically significant association between Ki67 and pCR was identified (*p*-value < 0.05). Furthermore, since patients belonging to the private DB underwent different therapy schemes, a variable outlining the performed therapy (therapy type) was also considered, but it did not show a significant association with pCR (*p*-value > 0.10). In our further analysis, when SVM classifiers exploiting clinical variables were designed, the variables that resulted as significantly associated with pCR for at least one of the two case studies were included. They were ER, PgR, HER2, grading and Ki67.

### 3.2. Evaluation Perfomance Achieved by the AI Model on Sagittal and Axial Baseline MRIs

The results achieved by the same AI approach on the training sets related to the two case studies are reported in Appendix A and represented in Table S1. A total of 29 and 28 features were selected as optimal features by applying all the steps of the AI method for both the public DB and the private DB. The proposed AI framework was then validated on two independent tests, one for each of the two case studies. Table 3 summarizes the results obtained from the independent tests by the models exploiting only clinical features, only F-merged features and clinical features in combination with the F-merged feature set.

The model, with input of the clinical variables alone, reached an AUC value of 58.2% and 56.0% and an accuracy of 64.4% and 59.1% for the public DB and the private DB, respectively. In this case, the specificity values were greater than the sensitivity values, similar to the results on the training sets (see Appendix A and Table S1). The usage of the F-merged feature set allowed us to improve the accuracy of the results. Overall, the best results were obtained when the clinical features were added to the F-merged features: an AUC value of 80.3% and 78.0%, an accuracy of 84.4% and 77.3%, a sensitivity of 69.2% and 71.4% and a specificity of 90.6% and 80.0% were achieved on the public DB and private DB, respectively. Slightly higher figures for the public DB might be related to the larger values of tumor size (see Table 1). Moreover, the public DB is composed of a greater number of patients with more homogenous characteristics, such as the NAC scheme undergone. However, more balanced results in terms of all the evaluation metrics were achieved on the private DB. As a final result, the AI method proved itself to be robust at interpreting various MRI acquisition views.

## 4. Discussion

With the increased use of deep learning techniques in all areas of the biomedical field [38,39], several attempts to solve the early prediction of pCR to NAC in breast cancer patients have been proposed. Liu et al. [40] used the first post-contrast pre-treatment MRI examinations from 131 patients (40 pCR; 91 non-pCR) of the I-SPY1 TRIAL public database to design a CNN-based method to predict pCR. As a result, a mean AUC value of 72% was returned. Ravichandran et al. [26] trained a CNN that made use of pre-contrast and post-contrast pre-treatment MRI scans referred to 133 patients of the I-SPY1 TRIAL public database. The method was validated on 33 patients, reaching an AUC value of 70% and 77% when the post-contrast MRI examinations were considered alone or in conjunction with the pre-contrast MR images, respectively. In this study, we developed and applied an AI framework to two different case studies based on MRI examinations acquired according to varying orientations (sagittal and axial). To the best of our knowledge, all previously developed models have been tailored for a specific protocol (axial or sagittal acquisition view). No investigation into the robustness of AI methods in various case studies with reference to different MRI acquisition views has previously been carried out for the early prediction of pCR to NAC in breast cancer patients. This aspect can be crucial to prove the generalizability of the method on data provided by multiple cancer institutions. As demonstrated elsewhere [26,41], the addition of clinical variables could contribute to improving the performances achieved by using an AI approach. By combining the CNN-features with the clinical variables, the overall performances were stable at varying MRI acquisition views: an AUC value of 80.3% and 78.0% was achieved on the independent tests conducted for the public DB and the private DB, respectively. Among the clinical variables, ER, PgR and HER2 had a significant association with pCR for both case studies. The variable Ki67 was significantly associated with pCR only for patients undergoing the AC + taxane therapeutic scheme. We can observe how the mean T appears differently between the two datasets and across the two classes (pCR and non-pCR, see Table 1). This is because of the timelines of the two case studies: while several years ago NAC was only used for patients with specific requirements in terms of tumor diameter (e.g., greater than 3 cm), nowadays, NAC has become a standard therapy in clinical practice and is received by the majority of patients regardless of their tumor size, and especially if the tumor is categorized as Triple-Negative or HER2+ [42,43,44]. Despite the promising results, our study has some limitations. The cohort of patients used for our analysis was relatively small, especially in reference to the patients on the private DB that were referred to our Institute. Moreover, an expected drop of performances in passing from the training sets to the independent tests was observed: since there was a limited number of patients belonging to the training sets, a feature selection was more prone to overfitting. To overcome this limitation in a future extension of the study, the robustness and flexibility of our findings will be proven on a larger cohort of patients across multiple institutions and protocols. Manifold are the possible extensions of this work. Future works will be focused on a volumetric analysis by jointly involving all slices of the MRI examinations [45]. An improvement in the prediction performances could be obtained by developing an AI model which could integrate multimodal data, including pre-treatment clinical information joined with features extracted from several kinds of images, such as pre-treatment MRI examinations before and after injection, as well as diffusion weighted images. The fusion of the information from different sources, and especially from different kinds of images, through AI models has already been proven as promising [46]. Each datum of a different nature contains part of the description of the same objects of interest. By combining together all these data, hidden complex relationships between the different modalities can be recognized by AI models [47,48].

## 5. Conclusions

In conclusion, we proposed an AI framework based on deep learning that has revealed itself to be able to give an early prediction of NAC outcome in terms of pCR in breast cancer patients undergoing NAC. In clinical practice, this aspect is essential for medical figures to promote personalized tools with the aim of contributing to the optimal selection of treatment and therapeutic options. Despite several efforts having been made in the state-of-the-art to give an early prediction of pCR to NAC, there is a lack of generalizable methods, rather than approaches applied only on sagittal or axial MRI examinations. Hence, more generalizable methods need to be developed with the aim of being easily utilized in large multi-institutional studies where the acquisition protocols may differ across diverse institutions. In this work, we wanted to fill this gap. The proposed approach appeared robust at various acquisition views of the MRI examinations performed prior to treatment: both axial and sagittal. This work represents a first effort towards the implementation of a more generalizable approach that can overcome protocol-specific methods, so that it can be utilized in multi-center studies exploiting diverse imaging acquisition protocols.

## Figures and Tables

**Table 1:** Patient characteristics.

Public DB			Private DB		
	pCR	Non-pCR		pCR	Non-pCR
Overall	42 (28%)	109 (72%)	Overall	22 (30%)	52 (70%)
Age (years)			Age (years)		
Mean ± std	46.81 ± 8.59	49.06 ± 9.15	Mean ± std	51.55 ± 12.72	52.02 ± 12.39
T (mm)			T (mm)		
Mean ± std	75.86 ± 36.39	65.24 ± 27.66	Mean ± std	35.78 ± 20.95	36.05 ± 18.13
Grading			Grading		
G1	0 (0%)	8 (7.4%)	G1	1 (4.5%)	1 (1.9%)
G2	12 (28.6%)	60 (55.0%)	G2	1 (4.5%)	16 (30.8%)
G3	27 (64.3%)	41 (37.6%)	G3	20 (91.0%)	30 (57.7%)
NA	3 (7.1%)	0 (0%)	NA	0 (0%)	5 (9.6%)
ER			ER		
Negative	29 (69.0%)	38 (34.9%)	Negative	12 (54.5%)	13 (25.0%)
Positive	13 (31.0%)	71 (65.1%)	Positive	10 (45.5%)	38 (73.1%)
NA	0 (0%)	0 (0%)	NA	0 (0%)	1 (1.9%)
PgR			PgR		
Negative	34 (81.0%)	49 (45.0%)	Negative	17 (77.3%)	21 (40.4%)
Positive	8 (19.0%)	60 (55.0%)	Positive	5 (22.7%)	30 (57.7%)
NA	0 (0%)	0 (0%)	NA	0 (0%)	1 (1.9%)
Ki67			Ki67		
Negative	2 (4.8%)	4 (3.7%)	Negative	0 (0%)	0 (0%)
Low	2 (4.8%)	28 (25.7%)	Low	0 (0%)	2 (3.8%)
Intermediate	7 (16.7%)	32 (29.4%)	Intermediate	4 (18.2%)	15 (28.8%)
High	20 (47.6%)	34 (31.1%)	High	18 (81.8%)	34 (65.5%)
NA	11 (26.1%)	11 (10.1%)	NA	0 (0%)	1 (1.9%)
HER2			HER2		
Negative	24 (57.1%)	86 (78.9%)	Negative	10 (45.5%)	35 (67.3%)
Positive	17 (40.5%)	22 (20.1%)	Positive	12 (54.5%)	16 (30.8%)
NA	1 (2.4%)	1 (1.0%)	NA	0 (0%)	1 (1.9%)

In the brackets, percentage values are specified. The abbreviation NA indicates missing values.

**Table 2:** Statistical analysis on clinical features.

Variable	Type	DB	*p*-Value
Age	Continuous	public	0.1673
		private	0.8805
T	Continuous	public	0.2508
		private	0.8097
ER	Categorical (binary)	public	1.2 × 10^−4^
		private	0.0164
PgR	Categorical (binary)	public	4.9 × 10^−5^
		private	0.0046
HER2	Categorical (binary)	public	0.0087
		private	0.0617
Grading	Categorical	public	1.4 × 10^−4^
		private	0.0286
Ki67	Categorical	public	0.0116
		private	0.3494
		private (over patients AC + tax)	0.0995

The Wilcoxon–Mann–Whitney test was performed for continuous features, whereas Spearman rank test was used for categorical features. A result was considered statistically significant when the *p*-value was less than 0.10.

**Table 3:** Summary of the performances achieved by the pCR prediction models in terms of AUC.

Set	Model	N. Features	AUC	Acc.	Sens.	Spec.
Public DB Independent test: 45 patients (13 pCR)	Clinical	5	58.2%	64.4%	53.6%	68.8%
	F-merged	29	75.0%	73.3%	**69.2%**	75.0%
	F-merged + clinical	34	**80.3%**	**84.4%**	**69.2%**	**90.6%**
Private DB Independent test: 22 patients (7 pCR)	Clinical	5	56.0%	59.1%	42.9%	66.7%
	F-merged	28	72.4%	**77.3%**	57.1%	**86.7%**
	F-merged + clinical	33	**78.0%**	**77.3%**	**71.4%**	80.0%

Accuracy (Acc.), Sensitivity (Sens.), and Specificity (Spec.) on the independent tests of the public DB and private DB. The number of features comprising each model is also reported. The best results achieved for each of the evaluation metrics are indicated in bold.

## Data Availability

Data images refer to the public database that is part of The Cancer Imaging Archive (TCIA) at the following link: https://wiki.cancerimagingarchive.net (accessed on 25 November 2021). Data obtained from the private database are available on request from the corresponding author. These data are not publicly available because they are the propriety of I.R.C.C.S.—Istituto Tumori ‘Giovanni Paolo II’—Bari, Italy.

## Supplementary Materials

The following supporting information can be downloaded at: https://www.mdpi.com/article/10.3390/jpm12060953/s1, Table S1: Summary of the performances achieved by the pCR prediction models in terms of AUC, Accuracy (Acc.), Sensitivity (Sens.), and Specificity (Spec.) on the training sets of the public DB and private DB. The number of features comprising each model is also reported. The best results achieved for each of the evaluation metrics are indicated in bold.

## Appendix A

### Appendix A.1. Technical Details about the Proposed AI Framework

The main steps of the proposed AI framework are summarized in the following.

#### Appendix A.1.1. Feature Extraction

Artificial intelligence and in particular deep learning models based on Convolutional Neural Networks (CNNs) have been successfully adopted as classifiers to solve several tasks in medicine [41,47,48] which involve image analysis. However, they are also used to extract features from the raw images. Here, we exploited a pre-trained CNN, called AlexNET [34], to automatically extract features from ROIs related to baseline MRIs. ROIs were input directly to the CNN architecture, which was pre-trained on ImageNet, a dataset of over one million of natural images designed for general object recognition. In this study, the knowledge learned by the network during such a training phase was transferred on the MRI exams. These features were then utilized to discriminate “early on” if a patients achieved pCR or not (pCR vs. non-pCR). The usage of a pre-trained network was preferred here since both the public and private DBs were composed by a number of images that was not sufficient to train a customized network [28,47]. The used network consists of 25 layers in total, including 5 convolutional layers, 3 pooling layers and 3 fully connected layers [34], where convolutional layers represent features at different level of input representation and abstraction, whereas pooling layers make features invariant to truncation, occlusion and translation [11]. Starting layers capture low-level features, which are features related to the local structure of the image such as lines, edges and blobs. Deeper layers return more complex and abstract features related to global characteristics of the image. They are obtained by combining low-level features. In this work, features from the three pooling layers of the network (called *pool1*, *pool2* and *pool5*) were automatically extracted to utilize the information within the feature maps at different resolutions and abstractions (see Figure 2 in the main text). Each input image that was the ROI extracted from the pre-processing phase was then resized to 227 × 227 pixels, since the pre-trained network required input data of this size. In details, the output of *pool1 layer* had dimensions of 27 × 27 × 96. A single 69,984-length vector was finally obtained by flattening the output. The *pool2 layer* had an output with dimensions of 13 × 13 × 256 that was flattening to a single 43,264-length vector, whereas the *pool5 layer* returned an output of 6 × 6 × 256 that was transformed in a 9216-length vector.

#### Appendix A.1.2. Stratified Feature Selection

A Stratified Feature Selection (SFS) was performed to finally choose a set of most stable features in correspondence of each of the three pooling layers used for feature extraction. Such a process was conducted on 70% of the overall data for the two databases separately (training sets). The remaining 30% of data for both datasets were employed as independent tests to validate the proposed model. The SFS consists of two main stages. As the first stage, a feature selection procedure was implemented according to a Leave-One-Out (LOO) cross-validation scheme to obtain a feature vector for each left-out patient of the training set (see the 2b in the main text). When a patient was left out, an iterative procedure based on stacking 10 subsets composed by the remaining 90% of the data, which were randomly selected, was performed. For each stacked subset of each iteration, important features were selected by applying two feature selection techniques via a cascade mechanism. A first well-known feature selection method, such as the non-parametric statistical Wilcoxon–Mann–Whitney test [32], evaluated the discrimination power with respect to the class (pCR or non-pCR) of each feature separately. The features that were resulted as statistically significant (with *p* < 0.001 for the *pool1* and *pool2* layers and with *p* < 0.05 for the *pool5* layer) became the input of a second feature selection algorithm, namely, Random Forest (RF), that chose a feature as important by comparing its importance with respect to all the features at disposal. In our simulations, the configuration of RF counted 100 trees. The features selected for each of the stacked subsets were unified in a single set. As shown in Figure 2 in the main text, at one iteration, one subset of features was associated. For each patient left out, the subsets of features obtained from the following iterations were intersected among each other thus finally obtaining a single feature set for that patient. The number of iterations (*n*) was experimentally estimated as *n =* 20. As the second stage of the SFS procedure, a single feature vector that was meaningful for all the patients of the training set was determined for each of the three pooling layers of the network, separately. Given a layer, such a feature vector was computed by intersecting all the subsets of features (one for each patient) that were outcome of the previous stage of the SFS procedure. These last feature sets were called as *F-pool1*, *F-pool2* and *F-pool5* in correspondence of *pool1*, *pool2* and *pool5* layers. Finally, the three feature sets contained the optimal features, i.e., the most stable features, that were less susceptible to variations in training samples. Several Support Vector Machine (SVM) classifiers were designed on these set of patients and a leave-one-out cross validation procedure on the training sets was implemented. A first classifier took in input the features belonging to *F-pool1*; a second classifier used the features of the *F-pool2* set; a third classifier employed the *F-pool5* as set of features. Another SVM classifier exploiting the F-merged set was designed. Finally, some clinical variables were added to the F-merged and exploited to build one further SVM classifier. The performances of these classifiers were compared with an SVM classifier using the clinical variables alone.

#### Appendix A.1.3. Classification

The power of the optimal selected features in discriminating “early on” patients who achieved pCR or not was assessed by separately training SVM classifiers on the sets of patients from the private DB and the public DB, which were used to identify the optimal subset of CNN features extracted for each layer. The classifiers were then validated on independent tests from the public and the private DB, respectively. A feature concatenation was conducted to merge the optimal features related to the three different layers: an optimal set of features, named F-merged, was constructed and then used to train another SVM classifier. Moreover, some clinical variables were evaluated alone or in addition to the F-merged. In correspondence, two diverse SVM classifiers were designed. Finally, clinical variables contain a few missing data. Such missing data were estimated by means of a proximity technique. For the training and test sets separately, the proximity technique allows us to replace the missing data of a patient with the respective data values referred to the patient without missing features with the closest HR and HER2 values: the Euclidean distance among the HR and HER2 values of the patients with missing data and completed data was computed.

### Appendix A.2. Results on Training Sets

Here, we present the results achieved by the same AI approach on the training sets related to the public DB and the private DB, respectively. They are reported in Table S1. The model exploiting clinical variables achieved comparable results on the two case studies (see the rows with clinical model in Table S1). An AUC value of 51.0% and 59.8% and an accuracy of 58.5% and 55.8% were achieved on the public DB and the private DB, respectively. Overall, the specificity assumed a higher value than sensitivity in both cases. The models built by using the optimal CNN-features extracted by the sagittal and axial MRIs, referred to the public and private DBs, respectively, were evaluated. The number of features contained in the feature sets in correspondence of the three layers is reported in Table S1. Features from the three pooling layers (*pool1*, *pool2* and *pool5*) of the network were automatically extracted to utilize the information within the feature maps at different resolutions and abstractions. Starting layers capture low-level features, which are features related to the local structure of the image such as lines, edges and blobs. Deeper layers return more complex and abstract features related to global characteristics of the image that are obtained by combining low-level features. The highest number of features was obtained for the *pool2* layer (13 features) and the *pool1* layer (12 features) in the case of the public DB and private DB, respectively. The lower number of features was returned for the *pool5* layer with respect to both DBs (6 features for the public DB and 5 features for the private DB, respectively). On the sagittal MRIs of the public DB, the models exploiting the CNN-extracted features belonging to the sets *F-pool1* and *F-pool5* reached almost comparable performances in terms of AUC values (i.e., AUC values of 82.6% and 81.7% respectively). Among the models employing the CNN-features extracted from the three layers separately, the highest specificity value (96.1%) was achieved by the model using the *F-pool2*. On the axial MRIs of the private DB, while the highest AUC value was achieved by using *F-pool1* (82.7%), the best accuracy was referred to the model exploiting *F-pool2* (84.6%). Merging the features belonging to *F-pool1*, *F-pool2* and *F-pool3* in a single set, i.e., F-merged, allowed it to globally outperform the previous models, especially in terms of AUC values: an AUC value of 86.7% and 86.1% was obtained on the public DB and private DB, respectively. Apart from the specificity value, the best performances were reached by combining the features belonging to the F-merged with the clinical features above mentioned in the previous subparagraph: an AUC value of 92.9% and 88.4%, an accuracy of 82.1% and 88.5% were achieved on the public DB and private DB, respectively.
