# Supplementary material for: Robustness Evaluation of a Deep Learning Model on Sagittal and Axial Breast DCE-MRIs to Predict Pathological Complete Response to Neoadjuvant Chemotherapy
Source: J Pers Med. 2022 Jun 10;12(6):953. doi: 10.3390/jpm12060953 (PMC9225219; doi:10.3390/jpm12060953)
Supplement: Supplementary file 1 [file jpm-12-00953-s001.zip › jpm-1674607-supplementary.pdf]

Table S1. Summary of the performances achieved by the pCR prediction models in terms of AUC, Accuracy (Acc.), Sensitivity (Sens.), and Specificity (Spec.) on the training sets of the public DB and private DB. The number of features composing each model is also reported. The best results achieved for each of the evaluation metric are indicated in bold.

Set	Model	N. features	AUC	Acc.	Sens.	Spec.
**public DB training set: 106 patients (29 pCR)**	clinical	5	51.0%	58.5%	31.0%	68.8%
	F-pool1	10	82.6%	71.7%	68.9%	72.7%
	F-pool2	13	73.7%	79.2%	34.5%	**96.1%**
	F-pool5	6	81.7%	77.4%	62.1%	83.1%
	F-merged	29	86.7%	81.1%	62.1%	88.3%
	F-merged + clinical	34	**92.9%**	**82.1%**	**75.9%**	84.4%
**private DB training set: 52 patients (15 pCR)**	clinical	5	59.8%	55.8%	13.3%	73.0%
	F-pool1	12	82.7%	80.7%	73.3%	83.8%
	F-pool2	11	74.6%	84.6%	66.7%	**91.9%**
	F-pool5	5	73.2%	76.9%	53.3%	86.5%
	F-merged	28	86.1%	80.8%	86.7%	78.4%
	F-merged + clinical	33	**88.4%**	**88.5%**	**86.7%**	89.2%
